# An Early Diagnostic Clue for *COL18A1-* and *LAMA1-*Associated Diseases: High Myopia With Alopecia Areata in the Cranial Midline

**DOI:** 10.3389/fcell.2021.644947

**Published:** 2021-06-25

**Authors:** Panfeng Wang, Xiaoyun Jia, Xueshan Xiao, Shiqiang Li, Yuxi Long, Mengchu Liu, Yongyu Li, Jun Li, Yan Xu, Qingjiong Zhang

**Affiliations:** ^1^State Key Laboratory of Ophthalmology, Zhongshan Ophthalmic Center, Sun Yat-sen University, Guangzhou, China; ^2^Guangdong Provincial Key Laboratory of Reproductive Medicine, Reproductive Medicine Center, The First Affiliated Hospital, Sun Yat-sen University, Guangzhou, China

**Keywords:** alopecia areata, cranial midline, syndromic high myopia, molecular genetics, diagnostic techniques

## Abstract

**Background:**

High myopia with alopecia areata in the occipital region has been observed in patients with Knobloch syndrome caused by *COL18A1* mutations. This study investigated other possible genetic causes of high myopia in patients with alopecia areata in the cranial midline.

**Methods:**

Six patients with early onset high myopia and alopecia areata in the cranial midline were recruited. Targeted high-throughput sequencing was performed on the proband’s DNA to detect potential pathogenic variants. Cosegregation analysis was performed for available family members. Minigene assay and RNA Sequencing were used to validate the abnormality of possible splicing change and gross deletion. Ophthalmological and neuroimaging examinations were performed.

**Results:**

Eight novel and one known loss-of-function mutants were detected in all six patients, including a gross deletion detected by RNA sequencing. Four *COL18A1* mutants in three patients with scalp leisure in the occipital region; and five *LAMA1* mutations in three patients with scalp leisure in the parietal region. Further assessments indicated that patients with *COL18A1* mutations had Knobloch syndrome, and the patients with *LAMA1* mutations had Poretti–Boltshauser syndrome.

**Conclusion:**

Our study found that early onset high myopia with midline alopecia areata could be caused not only by mutations of the *COL18A1* gene but also by mutations in the *LAMA1* gene. To our knowledge, we are the first to observe scalp defects in patients with *LAMA1* mutations. High myopia with alopecia areata in the cranial midline could be treated as an early diagnostic clue for ophthalmologists to consider the two kinds of rare diseases.

## Introduction

Myopia, particularly high myopia, could occur independently or as a feature in hundreds of ocular and systemic syndromes. Myopic syndromes have a wide range of clinical manifestations that affect patients’ daily lives in various ways, from mild discomfort to even death shortly after birth or in childhood. The nervous system is one of the most common parts of the body involved in these syndromes in addition to myopia; when affected, it can present with typical signs such as intellectual disability, microcephaly, cerebellar hypoplasia, special facial features, and hearing loss. These syndromes are usually rare and complex and include Coffin–Siris syndrome 1 (OMIM_135900) (Hoyer et al., 2012); Donnai–Barrow syndrome (OMIM_222448) (Kantarci et al., 2007); DOOR syndrome (deafness, onychodystrophy, osteodystrophy, mental retardation and seizures; OMIM_220500) (James et al., 2007); Hamamy syndrome (OMIM_611174) (Hamamy et al., 2007); Knobloch syndrome (KNO; OMIM_267750) (Passos-Bueno et al., 1994; Sertie et al., 2000); muscular dystrophy-dystroglycanopathy (congenital with brain and eye anomalies), type A, 10 (OMIM_615041) (Vuillaumier-Barrot et al., 2012); Pitt–Hopkins syndrome (OMIM_610954) (Whalen et al., 2012); Poretti–Boltshauser syndrome (PBS; OMIM_615960) (Aldinger et al., 2014; Micalizzi et al., 2016); Rubinstein–Taybi syndrome 2 (OMIM_613684) (Bartsch et al., 2010); Schuurs–Hoeijmakers syndrome (OMIM_615009) (Schuurs-Hoeijmakers et al., 2012); Shprintzen–Goldberg syndrome (OMIM_182212) (Doyle et al., 2012); and Temtamy syndrome (OMIM_218340) (Zahrani et al., 2013). Because high myopia might be an early feature in these syndromes or due to unawareness of the atypical manifestations of other signs, it is challenging for ophthalmologists to recognize these myopic syndromes, especially in children under 10 years of age. It would be helpful if certain signs could be obtained by physical examination to help doctors consider myopic syndromes with nervous system involvement, as is possible with Marfan syndrome.

KNO, with a prevalence estimated to be less than 1/1,000,000 (Orphanet, 2020a), is an autosomal recessive developmental disorder characterized by typical eye abnormalities with occipital skull defects. Linkage analysis successfully identified that KNO1 is mainly caused by mutations in the *COL18A1* gene (OMIM_120328) (Sertie et al., 2000), which plays a role in embryonic eye development and neural tube closure (Aikio et al., 2013; Banerjee et al., 2013). The eye abnormalities included high myopia, cataracts, dislocated lenses, vitreoretinal degeneration, and retinal detachment, while the occipital skull defect ranges from encephalocele to occipital bone fenestrae, meningocele, and cutis aplasia. Other rare phenotypes, including nervous system defects and missing nails, have also been reported (Keren et al., 2007; Williams et al., 2008; Ackerman et al., 2012; Abouelhoda et al., 2016). Congenital midline scalp defects have been described for most patients, including small areas of alopecia with a flat, wine-color hemangioma, occipital cutis aplasia (Kliemann et al., 2003), pigmented lesion in the occipital area (Balikova et al., 2020), midline occipital soft tissue swelling and alopecia hypotonia (Mahajan et al., 2010), and hair abnormalities (Williams et al., 2008; Hull et al., 2016). Physical examination of the skin of the scalp could be a fast and convenient way for ophthalmologists to detect signs for considering KNO and *COL18A1* mutations when treating children with high myopia.

In this study, we performed targeted high-throughput sequencing on patients with early onset high myopia with cutis aplasia in the skull to identify possible causative genes. In addition, we sought to determine whether a simple physical examination of the skin of the scalp could provide meaningful clues for further examination for myopic syndrome with nervous system involvement.

## Methods

### Patient Selection

This study was approved by the Institutional Review Board of the Zhongshan Ophthalmic Center. Unrelated probands with early onset high myopia were collected from the Zhongshan Ophthalmic Center as part of the project to identify genetic loci and genes for high myopia. Patients presenting with skull defects due to cutis aplasia not only in the occipital region but also extending to the cranial midline were recruited. Informed consent conforming to the tenets of the Declaration of Helsinki was obtained from the involved individuals or their guardians prior to the study. Genomic DNA was prepared from the venous blood of the patients and available family members. Standard ophthalmological examinations including visual acuity, refractive error examination after mydriasis, slit lamp, and fundus photography were performed on all probands and available family members. Electroretinogram (ERG), B-scan, X-ray, CT, and MRI were performed on selected patients when possible. The ERG response was recorded according to the International Society for Clinical Electrophysiology of Vision (ISCEV) standards Marmor et al. (1989).

### Mutation Screening and Analyses

Whole-exome sequencing (WES) or targeted exome sequencing (TES) was performed on genomic DNA from six probands to screen genetic defects. The WES or TES data were analyzed through multistep bioinformatics analysis as described previously (Li et al., 2015; Wang et al., 2019). The genes of target panel are listed in Supplementary Table 1. The possible influence of splicing change was predicted by software including Human Splicing Finder^1^ and Berkeley Drosophilia Genome Project (BDGP)^2^. Potential pathogenic variants (PPVs) were further classified according to the guidelines of the American College of Medical Genetics and Genomics (ACMG) (Richards et al., 2015); and variants of uncertain significance (VUSs), likely benign, and benign variants were ruled out. Sanger sequencing was used to confirm PPVs and to validate their cosegregation in available family members. The PPVs were described according to the nomenclature for sequence variations (Human Genome Variation Society) (den Dunnen and Antonarakis, 2000). The transcript, NM_130444.2, was used to nomenclature variants detected in *COL18A1*; and the transcript, NM_005559.4, was used to nomenclature variants detected in *LAMA1*.

### Splicing Change Confirmed by Minigene Assay

Three rounds of PCR were performed using nested primers (Supplementary Table 2): the first PCR was performed using genomic DNA (a total of two sets of DNA) as a template, with two pairs of primers, 49029-COL18A1-F and 51125-COL18A1-R (product length: 2,096 bp), as well as 49321-COL18A1-F and 50763-COL18A1-R (product length: 1,443 bp) with annealing temperature of 57°C, treating the products with size of 1,443 bp as a template. The second PCR was performed to produce human COL18A1 fragment covering exon 35 (145 bp)–intron 35 (79 bp)–exon 36 (74 bp)–intron 36 (406 bp)–exon 37 (129 bp), as follows: pcDNA3.1-COL18A1-*Kpn*I-F and pcDNA3.1-COL18A1-*Bam*HI-R as primers were used to produce pcDNA3.1-wt (wild type) fragment of 833 bp; pcDNA3.1-COL18A1-*Kpn*I-F and COL18A1-MUT-R as primers were used to produce mutant fragment 1; and COL18A1-MUT-F and pcDNA3.1-COL18A1-*Bam*HI-R as primers were used to produce mutant fragment 2. Finally, mutant fragments 1 and 2 were mixed together with ratio of 1:1 and were treated as template to amplify pcDNA3.1-mut (mutant fragment) of 798 bp (c.4259-28_4265del) with primers of pcDNA3.1-COL18A1-*Kpn*I-F and pcDNA3.1-COL18A1-*Bam*HI-R.

The PCR products pcDNA3.1-wt and pcDNA3.1-mut were purified (DNA Gel Extraction Kit, SIMGEN, Hangzhou, China) and inserted into the eukaryotic expression vector pcDNA3.1 using *Kpn*I/*Bam*HI to construct two sets of plasmids: pcDNA3.1-COL18A1-wt and pcDNA3.1-COL18A1-mut. Fragments were verified by Sanger sequencing.

The four kinds of recombinant vectors were transiently transfer into human embryonic kidney cells (HEK-293) and cervical cancer cells (HeLa) according to the manufacturer’s instruction (Rapid Plasmid Mini Kit, SIMGEN). The transfected cells were cultured for 48 h and then collected for transcript expression analysis.

TRIzol method (RNAiso PLUS, TaKaRa, Dalian, China) was used to extract total RNA from HEK-293 cells and HeLa cells. Reverse transcription polymerase chain reaction (RT-PCR) was carried out [HifairTM 1st Strand Cdna Synthesis SuperMix for qPCR (Gdna digester plus), YEASEN, Shanghai, China] to check the splicing method and verified through Sanger sequencing.

### RNA Sequencing

RNA sequencing was carried out by commercial company (Shanghai Cino Medical Laboratory Co., Ltd., Shanghai, China). Peripheral blood was collected in PAXgene Blood RNA Tube (BD Biosciences, San Jose, CA, United States), and RNA was isolated by MagMAX for Stabilized Blood Tubes RNA Isolation Kit according to the kit’s instruction. All RNA sample were measured for quantity and quality by NanodropOne (Thermo Fisher Scientific, Waltham, MA, United States) and Qseq400 (BiOptic, New Taipei City, Taiwan). Samples with RNA quality score (RQS) above 7 were proceeded with RNA-seq library preparation by the KAPA RNA HyperPrep Kit (Kapa Biosystems, Inc., Woburn, MA, United States) after depletion of rRNA with the KAPA RNA HyperPrep Kit (Kapa Biosystems). The total RNA input was 1 μg. Sequencing was performed on Illumina HiSeq 2000 instrument with PE150, and 15-G raw data were achieved per sample. The RNA sequencing data were checked for quality using FastQC and mapped and analyzed using the VIPER Snakemake pipeline (Cornwell et al., 2018). Briefly, VIPER aligns the files to the hg19 transcriptome using STAR, followed by differential expression analysis using DESeq2. Visualization and sashimi plot of the *COL18A1* gene were performed by Integrative Genomics Viewer (IGV) (Thorvaldsdottir et al., 2013). The abnormality mRNA of *COL18A1* was further validate by RT-PCR and Sanger sequencing after being amplified with temple of cDNA.

## Results

**FIGURE 1:**
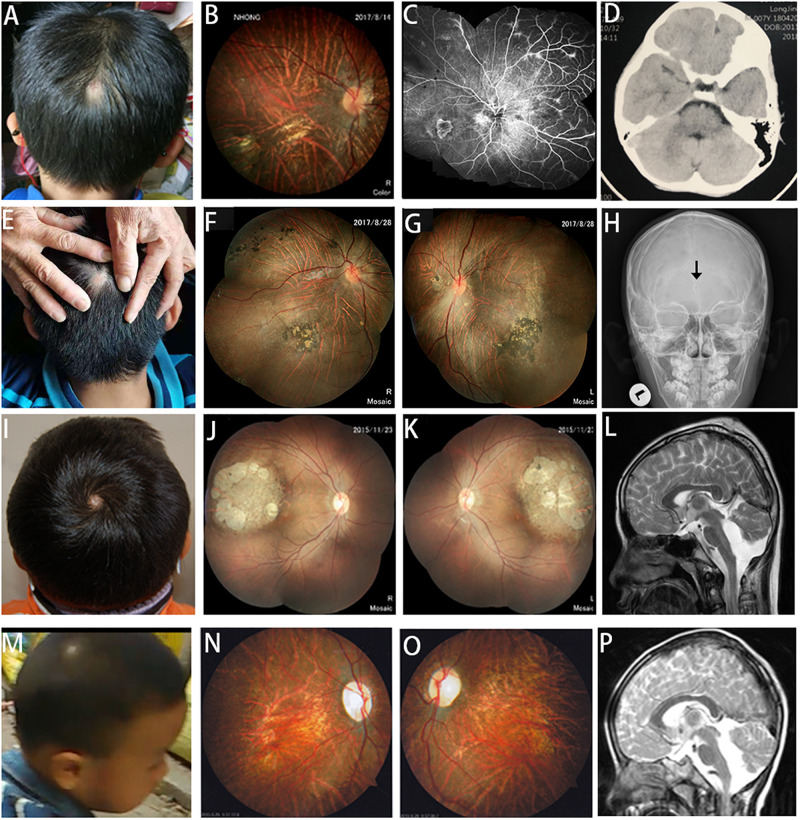
Scalp defects, fundus changes, and neuroimaging in patients with *COL18A1* and *LAMA1* mutations. **(A,E)** Alopecia areata appears in the midline of the occipital region of patients with *COL18A1* mutations. **(I,M)** Alopecia areata appears in the midline of the parietal region (in the center of hair whorl) in patients with *LAMA1* mutations. **(B,C,F,G,J,K,N,O)** In addition to the typical high myopia fundus leopard-like pattern, dysplasia of the papilla, and macular degeneration, characteristic fundus degeneration can be observed for all patients including optic degeneration, chorioretinal sclerosis and atrophy, foveal hypoplasia, atrophic patch with extra bone spicule accumulation, and loss of pigmentation temporal to the macula. **(C)** Fluorescein angiograms reveal vascular leakage in patient 14410. **(D)** MRI reveals a normal brain image for patient 14410. **(H)** Black arrow indicates a small hole in the occipital region of patient 14518 by X-ray. **(L,P)** Cerebellar dysplasia and obvious enlargement of the fourth ventricle are observed in patients 5176 and 7856.

A total of six unrelated probands (five males and one female) were recruited. The first complaint sign of strabismus was noticed in all patients, with an average age of 1.89 years (from 0.4 to 5). The average refractive error was –10.92 ± 1.83, and the best corrected vision was less than 0.2 (Table 1). All of them had skull defect from cutis aplasia in the cranial midline, three of which were located in the occipital region, while the rest were located in the parietal region (in the center of the hair whorl) (Figures 1A,E,I,M).

**TABLE 1:** Summary of clinical data in high myopia patients with alopecia areata in the cranial midline.

	14410	14518	20204	5176	7856	19618
Gene	*COL18A1*	*COL18A1*	*COL18A1*	*LAMA1*	*LAMA1*	*LAMA1*
Gender	Male	Male	Male	Male	Male	Female
Age at onset of disease (years)	5	2	4	2	0.5	0.4
First complain sigh	Strabismus	Strabismus	Nystagmus Strabismus	Strabismus	Strabismus	Strabismus
Age when examined	6	8	17	3	2.75	3
Visual acuity (R/L)	0.06/0.12	0.06/0.03	0.05/0.05	0.2/0.1	NA	NA
High myopia (R/L)	–10.25/–7.75	–11.50/–11.25	–18.00/–10.25	–11.50/–13.25	–12.25/–10.50	–15.00/–14.00
Anterior segment	(–)	(–)	Cataract OU	(–)	(–)	(–)
Optic disc	Peripapillary atrophy	Peripapillary atrophy	Peripapillary atrophy	Disc pallor	Disc pallor	Disc pallor
Leopard fundus	+++	+++	+++	+	+++	+++
Chorioretinal sclerosis and atrophy	+++	+++	+++	+	+++	+++
Macular atrophy	++	+++	+++	+++	+	+++
Pigment distribution	–	Center++/periphery++	Periphery++	Center+	–	Periphery++
Strabismus	+	+	+	+	+	+
Nystagmus	+	+	+	+	+	NA
Axial length (OD/OSmm)	NA	26.16/26.44	NA	26.32/26.01	NA	NA
ERG	NA	NA	Moderately reduced	Moderately reduced	Severe reduced	Moderately reduced
Scalp defect region	Occipital	Occipital	Occipital	Parietal	Parietal	Parietal
CSF leak	Transient	Transient	Transient	Denied	Denied	Denied
Skull	Normal	Occipital bone defect	Normal	Parietal bone defect	Normal	NA
MRI	Normal	NA	NA	Cerebellar dysplasia with enlarged fourth ventricle; cortical-subcortical cysts	Cerebellar dysplasia with enlarged fourth ventricle	NA
Other system defects	–	–	–	Accessary ear in the left	Motor delay and ataxia	Motor delay and ataxia

**NA, not available; ERG, electroretinogram; CSF, cerebrospinal fluid.**

**FIGURE 2:**
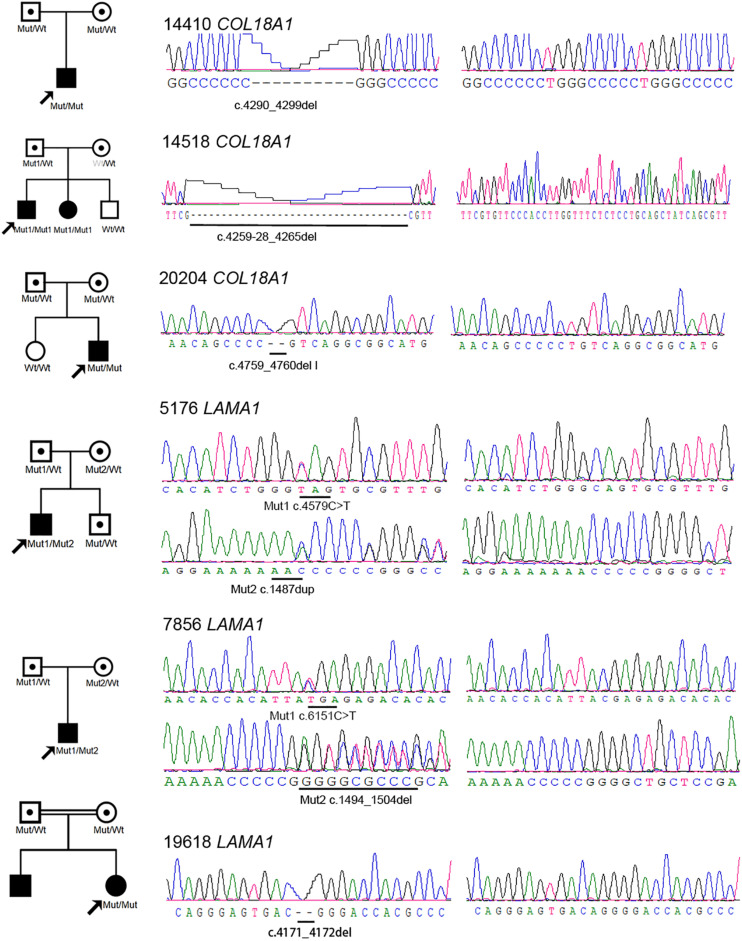
Pedigrees and sequences of eight novel loss-of-function mutations in the *COL18A1* and *LAMA1* genes. The genotypes of all probands and available family members are shown below each individual. The black symbols represent the affected individuals. The white symbols with black points represent carriers. Mut, mutation; wt, wild type. One allele of the wt in the mother of 14518 is marked as gray because it is a pseudo-wild type caused by gross deletion cross missing by Sanger sequencing.

Eight novel and one known potential pathogenic mutants were identified in these six probands (Table 2). Of these mutations, two were homozygous and one was compound heterozygous in the *COL18A1* gene, including c. [4290_4299del];[4290_42 99del][p.(Gly1016Glufs^∗^9)]; [(Gly1016Glufs^∗^9)], c.[4259-28_42 65del]; and c.[ex.32-36del] and a known mutation c.[4759_47 60del]; [4759_4760del]p.[(Leu1587Valfs^∗^72)]; [(Leu1587Valfs^∗^ 72)]. The remaining mutations were all located in the *LAMA1* gene, including c.[4579C>T]; [1487dup]p.[(Gln15 27^∗^)]; [(Asn496Lysfs^∗^15)], c.[6151C>T];[1494_1504del]p.[(Arg2 051^∗^)];[(Gly499Valfs^∗^8)], and c.[4171_4172del]; [4171_4172del]p.[(Arg1391Glyfs^∗^19)]; [(Arg1391Glyfs^∗^19)]. The mutation c.4259-28_4265del was predicted to activate a new cryptic Acceptor site and by HSF, and the confidence scores of the old Acceptor site were diminished from 0.88 to 0 before and after the mutation by FF. All eight mutations were predicted to result in complete loss of function (LOF) for the corresponding proteins. All mutations were confirmed with Sanger sequencing and segregated by disease in the available family members (Figure 2 and Table 2).

**TABLE 2:** Eight potential pathogenic mutations in *COL18A1* and *LAMA1* genes.

Patient ID	Gene	Position	Transcript	Nucleotide change	Exon	Status	Amino acid change	Effect	Origin	GenomAD-EAS	Sequencing strategy
14410	*COL18A1*	chr21:46925303_46925312	NM_130444.2	c.4290_4299del	E35/41	Hmz	p.(Gly1431Glufs*9)	Frameshift	Parents	None	TES
14518	*COL18A1*	chr21:46925244_46925278	NM_130444.2	c.4259-28_4265del	IVS34/41	Hmz	–	Splice site broken -86.23	Father	None	TES
		?	NM_130444.2	?	E32–E36	Het	Gross deletion		Mother	None	RNA sequencing
20204	*COL18A1*	chr21:46929996_46929997	NM_130444.2	c.4759_4760del	E39/41	Hmz	p.(Leu1587Valfs*72)	Frameshift	Parents	None	TES
5176	*LAMA1*	chr18:6999528	NM_005559.4	c.4579C > T	E32/63	Het	p.(Gln1527*)	Non-sense	Father	0	WES
		chr18:7038885		c.1487dup	E11/63	Het	p.(Asn496Lysfs*15)	Frameshift	Mother	5.45E–05	
7856	*LAMA1*	chr18:6978234	NM_005559.4	c.6151C > T	E43/63	Het	p.(Arg2051*)	Non-sense	Father	5.01E–05	WES
		chr18:7038868_7038878		c.1494_1504del	E11/63	Het	p.(Gly499Valfs*8)	Frameshift	Mother	None	
19618	*LAMA1*	chr18:7007226_7007227	NM_005559.4	c.4171_4172del	E29/63	Hmz	p.(Arg1391Glyfs*19)	Frameshift	Parents	1.50E–04	TES

TES, targeted exome sequencing; WES, whole-exome sequencing; *, Termination codon.

**FIGURE 3:**
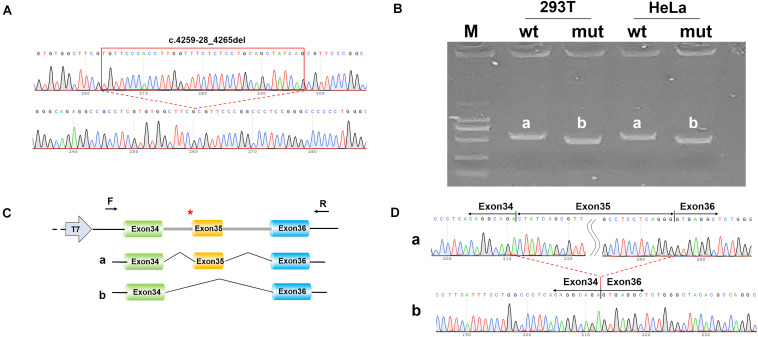
Alternative splicing assays in minigene. **(A)** Sequencing results of pcDNA3.1-wt (wild type) fragment (top) and pcDNA3.1-mut (mutant fragment) with c.4259-28_4265del of *COL18A1* (bottom). **(B)** Gel view of reverse transcription polymerase chain reaction (RT-PCR). The size of expression of pcDNA3.1-COL18A1-wt (band a) is larger than that of pcDNA3.1-COL18A1-mut in both 293T cell and HeLa cell. **(C)** Schematic diagram of minigene construction and schematic diagram of expression. The “a” represents the normal mRNA composed of exon 34, exon 35, and exon 36, while the “b” represents the aberrant mRNA with exon 36 skipped. * indicates the mutation position. **(D)** Sequencing results of the bands in **(B)**.

A minigene splicing assay was built to validate whether the splicing change detected in *COL18A1*, c.4259-28_4265del, affects splicing products (Figures 3A–D). A total of eight samples were harvested after 48 h of transfection. The gel view of the reverse transcription PCR showed that band b (mut) migrated faster than band a (wt), which meant that the mut fragment (274 bp) was smaller than that of wt (348 bp) (Figure 3B). DNA sequencing indicated that the wild-type minigene expressed normal mRNA composed of exon 34, exon 35, and exon 36; the mutant type minigene expressed a shorter mRNA composed of exon 34 and exon 36 (Figures 3C,D). The mutation c.4259-28_4265del damaged the Acceptor of Exon 35 and resulted in the skipping of exon 35. The result is consistent with the *in silico* prediction.

**FIGURE 4:**
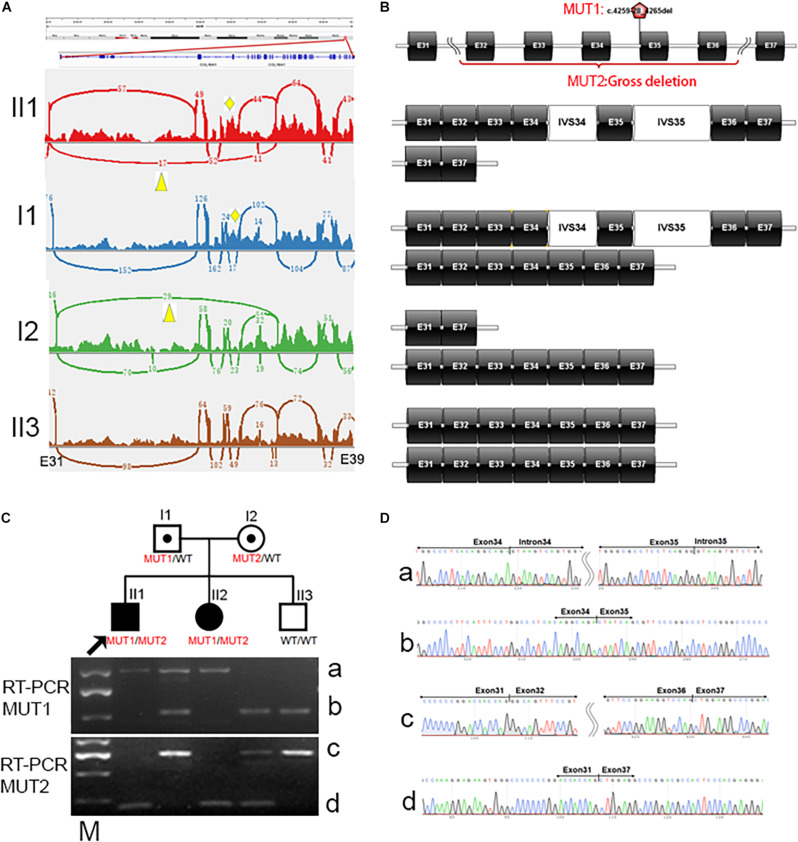
Identification of biallelic mutants for *COL18A1* in family 14518 by RNA sequencing. **(A)** Splicing-in of the pseudoexon, IVS34 and IVS35, is observed in proband II1 and his father I1, indicated by a yellow rhombus. Gross deletion crossing Exon 32 to exon 36 is observed in proband II1 and his mother, indicated by a yellow triangle. **(B)** Schematic diagram of four different kinds of transcript way between Exon 37 to exon 37 of *COL18A1* in proband, carriers, and normal of different genotypes. **(C)** The biallelic mutants is observed in RT-PCR amplicons from family 14518. MUT1: Compare with wildtype of band b with 525bp in II3, the aberrant band a is longer (1010 bp) due to splicing-in of the IVS34 and IVS35 (485 bp). MUT2: Compare with wildtype of band c with 724 bp covering exon 31-37 in II3, the aberrant band d is shorter (192 bp) caused by deletion of exon 32 to 36 (532 bp). **(D)** The aberrant bands are confirmed by Sanger sequencing. M: DL2000 DNA Marker with a brighter fragment at 750 bp.

In family 14518, cosegregation analysis showed that the proband and sister harbored homozygous mutation, c.4259-28_4265del, in *COL18A1*; the father harbored heterozygous mutation, while the mother and youngest brother were normal by Sanger sequencing. Since allele dropout has been ruled out by change amplify primers, it was reasonable to suspect potential heterozygous deletions involving one or more exons in a mother or independent uniparental disomy (UPD) that happened twice in this family from the father. To solve the question, RNA sequencing was performed on all five family members of 14518. By RNA sequencing, we identified a heterozygous intron inclusion event in proband II1 and his father I1 (Figures 4A,B). Different from *in silicon* prediction, the Acceptor splice site change variant, c.4259-28_4265del in *COL18A1*, created two splicing-in of the intronic segments of IVS34 and IVS35, leading to an an in-frame pseudoexon. Unexpectedly, another heterozygous aberrant exon deletion in proband II1 and his mother I2 was simultaneously observed (Figures 4A,B). The exon of exon 33 to exon 37 of *COL18A1* was skipped besides a normal transcript way. Both events were absent in II3, who is clinically normal. The results were further proved by electrophoresis (Figure 4C) and Sanger sequencing (Figure 4D). The gross deletion legitimately explained the phenotype that the mother seems normal in c.4259-28_4265del by Sanger sequencing. The proband harbored biallelic different mutants: one was c.4259-28_4265del, which was inherited from the father, and another was a gross deletion crossing exon 32 and exon 36, which was inherited from the mother. A similar situation happened in his younger sister, who also suffered from early onset high myopia and alopecia areata that appeared in the midline of the occipital regions, with encephalocele revealed by MRI when she was 3 months old. Cosegregation supported the pathogenetic biallelic mutants discovered in these families.

The ocular and systemic manifestations of the six probands are summarized in Table 1. Congenital cataracts were recorded in one patient (20204) with *COL18A1* mutant. In addition to the typical high myopia fundus characteristics, such as a leopard pattern, dysplasia of the papilla, and macular degeneration, characteristic fundus degeneration was noticed in all patients, including optic degeneration, chorioretinal sclerosis and atrophy, foveal hypoplasia, atrophic patch with extra bone spicule accumulation, and loss of pigmentation temporal to the macula (Figures 1B,C,F,G,J,K,N,O). Fluorescein angiograms exhibited vascular leakage in patient 14410 (Figure 1C). ERG indicates a moderate-to-severe decrease in cone and rod cells.

Skin thickening in the regions of alopecia areata was observed in all patients. Furthermore, alopecia areata appeared in the midline of the occipital regions of three patients with *COL18A1* mutations (Figures 1A,E). A small hole was found in the occipital bone of patient 14518 by X-ray (Figure 1H), while the other two had normal occipital bone. Transient leakage of liquid in the region of alopecia areata was reported by the parents of all three patients while they were infants. Normal brain images were exhibited on MRI for patient 14410 (Figure 1D). No extra abnormality was noticed in the other systems of all three patients. High myopia, defects in the occipital region, and *COL18A1* mutation supported a diagnosis of KNO for all three patients. For the three patients with *LAMA1* mutations, alopecia areata appeared in the midline of the parietal region (in the center of hair whorl) (Figures 1I,M). All parents denied observing transient leakage of liquid in the alopecia areata region. MRI examination was performed on patients 5176 and 7856, and the imaging results, including cerebellar dysplasia and obvious enlargement of the fourth ventricle, supported a diagnosis of PBS for both patients (Figures 1L,P). Developmental delay and ataxia were noticed in patients 7856 and 19618. An accessory ear on the left side was observed in patient 5176.

## Discussion

In the present study, pathogenic mutations were detected in six early onset high myopia patients with midline alopecia areata, and the causative genes included not only the *COL18A1* gene but also the *LAMA1* gene. RNA sequencing identified biallelic mutants for *COL18A1* including a variant introducing an in-frame pseudoexon, and a gross deletion missed by TES and Sanger sequencing. The patients shared characteristic fundus degeneration, although the systemic abnormalities were different, as they were caused by different genes. The scalp defect in the three patients with *COL18A1* mutations were in the occipital region, which is consistent with previous reports describing the defective area of patients with KNO caused by *COL18A1* mutants (Sertie et al., 2000; Aldahmesh et al., 2011). The scalp defect in the three patients with *LAMA1* mutations was in the parietal region (in the center of hair whorl). To our knowledge, there are no prior reports describing skull defects in humans with *LAMA1* mutations.

In family 14518, initially, we found a novel homozygous splicing change c.4259-28_4265del in the proband. The novel splice site variant (c.4259-28_4265del) is located at the 5’ splice site exon 35 of *COL18A1*, and the minigene assay showed that the mutation skipped exon 35 entirely, resulting in a new connection between exon 34 and exon 36. Although the pathogenesis of the splicing change was supported by minigene assay, a fault was observed in cosegregation result, of which the proband inherited this mutant from his father, but his mother seemed normal at this point by Sanger sequencing. To solve this question, RNA sequencing was performed on all five family members of 14518 and identified biallelic abnormality transcript event in *COL18A1* including IVS34 and IVS35 inclusion caused by c.4259-28_4265del, as well as an exons deletion caused by deletion of exon 32 to exon 36. There were four splicing changes and four gross deletions recorded by Human Gene Mutation Database (HGMD) (202002) and addressed as damaged mutation (DM) (Sertie et al., 2000; Keren et al., 2007; Suzuki et al., 2009; Aldahmesh et al., 2011; Retterer et al., 2016; Turro et al., 2020); and most of them were detected by WES or whole-genome sequencing (WGS), but none of them were identified or validated at the transcript level. The novel finding highlights the utility of RNA sequencing for the detection and interpretation of variants missed by the current routine diagnostic approach and provides a basis for genetic diagnosis of KNO.

The HGMD (Professional 202002) lists 33 DMs for the *COL18A1* gene and 35 DMs for the *LAMA1* gene; of these, 88% (29/33) in *COL18A1* and 91% (32/35) in *LAMA1* were nonsense, frameshift, splice site variants, and copy number variations (CNVs), all of which were predicted to result in LOF. In our study, all night mutants we detected were LOF including three frameshifts (c.4290_4299del, c.4759_4760del, and ex.32-36 deletion) and one splice site variant (c.4259-28_4265del) in *COL18A1*, and three frameshifts (c.1487dup, 1494_1504del, and c.4171_4172del) and two non-senses (c.4579C > T and c.6151 C > T) in *LAMA1*. In this study, the *COL18A1* mutations clustered at the C terminal, and the clinical manifestations of the patients with *COL18A1* mutations primarily consist of ocular abnormalities, including high myopia, fundus degeneration, and strabismus, while the system phenotypes were mild, as alopecia areata appeared in the midline of occipital region. The mutation, c.4759_4760del (p.L1172VfsX72) in proband 20204, was one hotspot and has been detected in different patients (Suzuki et al., 2009; Joyce et al., 2010; Aldahmesh et al., 2011; Aldahmesh et al., 2013). The phenotypes of patients with the c.4759_4760del (homozygous) mutations ranged from ocular abnormality without systemic defect (Aldahmesh et al., 2011), ocular abnormality and mild occipital cutis aplasia (Aldahmesh et al., 2013), to visual problems (glaucoma, lens dislocation, and retinal and corneal dystrophy), cerebellar ataxia, and cognitive deficiency (Paisan-Ruiz et al., 2009), to epilepsy without occipital defect (Aldahmesh et al., 2013). The position of mutation cannot explain the mild systemic phenotype in our study. Similar situations were also observed in the patients with *LAMA1* mutation, and more effort is necessary to identify possible phenotypic modifiers.

PBS, with a prevalence estimated to be less than 1/1,000,000, is an autosomal recessive cerebellar dysplasia syndrome caused by biallelic mutations in *LAMA1* (Orphanet, 2020b). The LAMA1 protein (laminin α1) is major component of the basement membrane and plays a role in cell adhesion, differentiation, proliferation, and migration. The defect of *LAMA1* will lead to PBS in human and embryonic lethality in mice because of multiple brain abnormalities (Heng et al., 2011). PBS is characterized by typical cerebellar dysplasia (100%) with cysts (91%) and abnormally shaped fourth ventricle (83%). Most of the patients revealed developmental delay as the first symptom at age below 6 months. The clinical features comprise non-progressive cerebellar ataxia (100%), cognitive function defect ranging from normal to intellectual disability (88%), and eye abnormalities including myopia (67%), ocular motor apraxia (67%), strabismus (54%), and retinopathy (46%) (Aldinger et al., 2014; Micalizzi et al., 2016). Rare phenotypes were also reported including dry skin, brain malformations, hypotonia, retinal vasculopathy, severe arthrogryposis, and tics (Supplementary Table 3). In contrast to typical PBS patients, the three unrelated patients with *LAMA1* mutations in our study visited an ophthalmology clinic for their strabismus problems when they were infants (2, 0.5, and 0.4 years old), at which point their high myopia and characteristic fundus degeneration were discovered. Neurodevelopmental deficits, such as motor delay, speech delay, or cognition impairment were mild or negligible. The classical neuroimaging changes in PBS were confirmed by MRI after *LAMA1* mutations were detected. Children with early onset high myopia might harbor other ocular or systemic symptoms despite a full assessment due to unawareness or atypical manifestation of other major signs. It is important that further examinations be performed for children with early onset high myopia to diagnose early possible myopic syndrome.

KNO and PBS are rare and highly clinical heterozygous genetic diseases. The occipital defects of KNO can range from mildly pigmented skin spot on the back of the head to alopecia areata in our study, and encephalocele, which forms part of the classical triad of symptoms. PBS also presents with a wide range of neurodevelopmental features, including non-progressive cerebellar ataxia, intellectual disability, and ocular abnormality. Their unique neuroimaging phenotypes are the gold standard when diagnosing the conditions. Manifestations of both syndromes outside of ocular are non-specific and hence do not provide useful clues of diagnosis, especially at onset. Ideally, a simple physical examination could provide meaningful clues to suggest further examinations for myopic syndrome involving the nervous system. In our study, we collected six patients with early onset high myopia and skull defects from cutis aplasia in the cranial midline, and 100% detected causative gene defects by targeted high-throughput sequencing in all patients. Further assessments indicated that the three patients with *COL18A1* mutations had KNO, and the three patients with *LAMA1* mutations had PBS. Alopecia areata in the occipital region or parietal region of the cranial midline, as observed by simple physical examinations, combined with the ocular abnormalities of early onset high myopia and characteristic fundus degeneration, dramatically delineated the two rare myopia syndromes with nervous system involvement, KNO, and PBS. Although the results were summarized from six unrelated families, the power of the association between the two manifestations and the two syndromes should be further evaluated in a larger cohort. The defects of the scalp could be considered as an early diagnostic clue of KNO or PBS for ophthalmologists when treating children with high myopia and alopecia areata in the cranial midline at eye clinics. Further function study is needed to clarify the pathogenesis between *LAMA1* mutation and alopecia areata in the occipital region or parietal region of the cranial midline.

## Data Availability Statement

The original contributions presented in the study are publicly available. This data can be found here: https://bigd.big.ac.cn/gsa-human/browse/HRA000519, and the accession number is HRA000519.

## Ethics Statement

The studies involving human participants were reviewed and approved by the Institutional Review Board of the Zhongshan Ophthalmic Center. Written informed consent to participate in this study was provided by the participants’ legal guardian/next of kin.

## Author Contributions

PFW participated in the entire process of research, data analysis, and wrote the first draft of the manuscript. XYJ, SQL, and XSX contributed to data collection and implementation of the research. YXL, MCL, YYL, JL, and YX performed the experimental process. QJZ guided the entire process in terms of theory and practice and revised the manuscript. All authors contributed to the article and approved the submitted version.

## Conflict of Interest

The authors declare that the research was conducted in the absence of any commercial or financial relationships that could be construed as a potential conflict of interest.

## Footnotes

1 http://www.umd.be/HSF3/

2 http://www.fruitfly.org
